# Digital Health Management During and Beyond the COVID-19 Pandemic: Opportunities, Barriers, and Recommendations

**DOI:** 10.2196/19246

**Published:** 2020-07-06

**Authors:** Becky Inkster, Ross O’Brien, Emma Selby, Smriti Joshi, Vinod Subramanian, Madhura Kadaba, Knut Schroeder, Suzi Godson, Kerstyn Comley, Sebastian J Vollmer, Bilal A Mateen

**Affiliations:** 1 University of Cambridge Cambridge United Kingdom; 2 The Alan Turing Institute London United Kingdom; 3 Central and North West London National Health Service Foundation Trust London United Kingdom; 4 Digital Mentality London United Kingdom; 5 Wysa Bangalore India; 6 Expert Self Care Bristol United Kingdom; 7 Centre for Academic Primary Care, University of Bristol Bristol United Kingdom; 8 MeeTwo London United Kingdom; 9 Warwick University Warwick United Kingdom; 10 Kings College Hospital London United Kingdom

**Keywords:** digital mental health, call to action, due diligence, data insights, COVID-19

## Abstract

During the coronavirus disease (COVID-19) crisis, digital technologies have become a major route for accessing remote care. Therefore, the need to ensure that these tools are safe and effective has never been greater. We raise five calls to action to ensure the safety, availability, and long-term sustainability of these technologies: (1) due diligence: remove harmful health apps from app stores; (2) data insights: use relevant health data insights from high-quality digital tools to inform the greater response to COVID-19; (3) freely available resources: make high-quality digital health tools available without charge, where possible, and for as long as possible, especially to those who are most vulnerable; (4) digital transitioning: transform conventional offline mental health services to make them digitally available; and (5) population self-management: encourage governments and insurers to work with developers to look at how digital health management could be subsidized or funded. We believe this should be carried out at the population level, rather than at a prescription level.

## Introduction

Although we maintain behaviors that limit the spread of the coronavirus disease (COVID-19), we must also ensure that measures are in place to prevent, or at least mitigate, the risks of individuals being harmed in other ways.

Before COVID-19, mental health and social services were already stretched. Depression is the second leading cause of disability worldwide, and by 2030, it is expected to be the leading contributor to the global burden of disease [1]. Efforts to contain the spread of COVID-19, including prolonged social distancing and self-isolation, may trigger or exacerbate social, mental, and physical health problems, such as anxiety, relationship breakdowns, domestic violence, substance abuse or withdrawal, and obesity [2-4]. This could be especially serious for those with comorbid medical and psychological conditions [5].

During the COVID-19 crisis, digital technologies have become a major route for accessing mental health care. Therefore, the need to ensure that these tools are safe and effective has never been greater. We raise five calls to action to ensure the safety, availability, and long-term sustainability of these technologies: (1) due diligence: remove harmful health apps from app stores; (2) data insights: use relevant health data insights from high-quality digital tools to inform the greater response to COVID-19; (3) freely available resources: make high-quality digital health tools available without charge, where possible and for as long as possible, especially to those who are most vulnerable; (4) digital transitioning: transform conventional offline mental health services to make them digitally available; and (5) population self-management: encourage governments and insurers to work with developers to look at how digital health management could be subsidized or funded. We believe this should be carried out at the population level, rather than at a prescription level.

## Action 1: Due Diligence

During this time of heightened health anxiety, poorly designed (or novelty) apps that give inaccurate or misleading information can do more harm than good, such as nudging inappropriate health-related behaviors, compromising access to necessary care, or having other negative unforeseen consequences. We call on app stores to take expert advice on which apps might undermine standard clinical practices and subsequently remove apps posing health risks. For example, there are “prank” blood pressure apps that give inaccurate, randomly generated readings to millions of users [6] and suicide and depression apps, which have been downloaded over 2 million times, that provide wrong or nonexistent information about suicide crisis helpline support [7].

## Action 2: Data Insights

We must consider data insights from high-quality digital health tools wherever possible to support the greater response to COVID-19. For example, an international team is working with multiple digital service providers to document the scale and nature of the mental health impact of COVID-19 [8]. We briefly highlight some changes in user interactions that have already been observed by Wysa [9] and MeeTwo [10] (Textbox 1). Wysa is an artificial intelligence–enabled chat-based, self-management resource that has been downloaded almost 2 million times worldwide, and is the current top recommendation of the National Health Service (NHS) and the Organisation for the Review of Care and Health Apps [11] for mental health and wellness. MeeTwo is a fully-moderated, anonymous peer support app currently supporting 25,000 young people aged 11-25 throughout the United Kingdom and is part of the NHS Apps Library. Another more specialized app called distrACT [12], which offers information and support to people who self-harm and may feel suicidal that is also included in the NHS Apps Library, has not observed substantial changes in patterns of user interactions at the time of writing.

**Quantitative and qualitative observations provided by the anonymous artificial intelligence–enabled chatbot Wysa and the anonymous moderated peer-to-peer support network MeeTwo, which demonstrate a visible shift in demand and change in use patterns during COVID-19.**

**Wysa (quantitative insights)**

- Wysa witnessed a 77% increase in new users during February and March 2020, as compared to the same period in 2019 [8].
- The proportion of users who referred to the coronavirus disease during therapist sessions increased week-by-week during March 2020, from 5% in the first week to 60% in the fourth week [8].

**MeeTwo (quantitative insights)**

- There were 27 suicidal posts between 8:30 AM and 8 PM on March 22, 2020 (48 hours after schools were closed), as compared to 406 suicidal posts in all of 2019 [8].
- There was a 95% increase in level 4 (severe risk) between March 20 and April 4, 2020, as compared to between December 20, 2019 and January 4, 2020 [8].
- There was a 116% increase in level 3 (high risk) posts between March 20 and April 4, 2020, as compared to between December 20, 2019, and January 4, 2020 [8].

**Wysa (qualitative insights)**

- Some of the causes of distress discussed in sessions included loss of access to a client’s therapists, medical support, or social worker due to lockdowns or social distancing; job loss during the coronavirus disease and not having health insurance coverage; loss of a loved one due to coronavirus disease–induced infection; fear of contracting the illness or loved ones contracting the illness; loss of sense of safety and security; loss of physical human support for clients with physical disabilities leading to more anxiety and fatigue; anxiety, hypervigilance, and loneliness from quarantining; increase in reporting of domestic violence incidents or abusive behavior due to quarantining, leading to less opportunities to access safe spaces or support; alcohol or nicotine withdrawals from lockdown impacting availability of addictive substances; and stigma around being a professional who comes in to contact directly or indirectly with a person who may have contracted the coronavirus disease [8].
- Clients have shared that they are in long waiting lines to get tested, that their own therapists or psychiatrists suddenly paused any upcoming appointments and are not available for consultations, which can all lead to panic as well as feelings of being abandoned by the system [8].

**MeeTwo (qualitative insights)**

- “My dad passed away due to the virus 2 days ago and honestly it just doesn't feel real yet” (April 1, 2020) [8].
- “camhs has passed me over to another service and because of covid-19 my counselling might have to be video calls at first and that makes me insanely uncomfortable. i hate calling people in my own family but a complete stranger??? no thank uuuuu” (March 3, 2020) [8].
- “I love this app but I wish I had someone to talk to in real life, I have no friends and my parents just don’t understand and literally don’t say anything so?? I was ALMOST gonna get a therapist but then corona virus happened and I bet the waiting list is going to be over a year long when everything goes back to normal ugh” (April 2, 2020) [8].

## Action 3: Freely Available Resources

The physical and mental demands on frontline care professionals puts them at increased risk of illnesses and death [13]. At this crucial time, we call for digital service providers to offer free tools and open access to services wherever possible. Some digital health providers have already made tools and resources freely available for health care workers, and some providers are offering certain resources freely for all of their users. This comes with a note of caution, however, as NHSX has received over 400 free platform offers, but many offerings are only for a short duration. A sudden removal of digital support can pose risks, such as creating dependency for patients, unclear expectations for care providers, or financial uncertainty. Therefore, we call on companies to strive to offer support for the full duration of need, which may extend beyond the immediate public health crisis. Furthermore, future approval of apps for health care use might take into consideration whether the digital tool could be made freely available during public health crises, and beyond, as needed.

## Action 4: Digital Transitioning

COVID-19 has led to nonessential staff working from home. NHSX recently published guidance to relax the rules around using digital communication tools to maintain therapeutic connections with patients. We call for training in digital health support to be incorporated into the curriculum for health professionals. We also call for conventional supportive services to be made available digitally, for example, sustaining services like Improving Access to Psychological Therapies (IAPT) given its reduced capacity, partly due to the redeployment of staff to newly established crisis care lines. As an example of this digital transitioning process, the Central and North West London Mental Health NHS Trust has established a protocol of good practice to transition from face-to-face art therapies to tele-art psychotherapies that also monitor physical care as part of the therapy [14].

## Action 5: Population Self-Management

We need more reimbursements or subsidizations for cost-effective preventive and self-management interventions for mental health at the population level rather than at a prescription level. A triage phone call to IAPT services costs approximately £100 (approximately US $125). Before COVID-19, two-thirds of callers were reported as inappropriate referrals [15]. Technologies that can support self-management and self-assessment could save governments money while directing expensive medical resources to those who need them most.

To summarize, we have identified some pressing issues, with actions that could address them; however, these are by no means comprehensive and come with uncertainties and risk (eg, gaining consensus as to what constitutes a list of acceptable apps, maintaining data security, and obtaining patient consent). Other effective and safe digital services could also be rapidly implemented (eg, virtual visiting for loved ones, especially for end-of-life patients). We also encourage stakeholders to consider the importance of managing screen time (eg, using voice recognition). Digital health is a priority area for widening reach and providing timely assistance. At a time of immense strain, we must supplement health care systems with digital health management support, and we must strive to reduce the risks that create additional burdens.

## Abbreviations

COVID-19: coronavirus disease
IAPT: Improving Access to Psychological Therapies
NHS: National Health Service
